# Azimuthal sensitivity and spatio-temporal decimation of data from distributed acoustic sensing on submarine cables for offshore earthquake early warning systems

**DOI:** 10.1038/s41598-025-29234-5

**Published:** 2025-11-25

**Authors:** Jack Lee Smith, Mohammad Belal, Karen Lythgoe, Carl Spingys, Jennifer Ward Neale, Davide Embriaco, Andrew Curtis

**Affiliations:** 1 School of Geosciences, University of Edinburgh, Edinburgh, UK; 2 National Oceanography Centre, Southampton, UK; 3 School of Electronics and Computer Science, University of Southampton, Southampton, UK; 4 Istituto Nazionale di Geofisica e Vulcanologia (INGV), Rome, Italy

**Keywords:** Distributed acoustic sensing, Earthquakes, Early warning, Angular resolution, Body waves, Natural hazards, Ocean sciences, Solid Earth sciences

## Abstract

Offshore earthquakes can cause widespread destruction due to directly propagating seismic vibrations and/or the generation of tsunamis. Early warning of offshore earthquakes is vital, but their location complicates identification of clear early warning (EW) signatures. Distributed acoustic sensing (DAS), applied to offshore fibre-optic cables offers the prospect to improve early warning of offshore events. We use a DAS dataset acquired from a 30 km submarine cable spanning a complex bathymetry, offshore Sicily, to contribute to a proof-of-concept using five regional earthquakes to investigate factors impacting incident body wave detection capabilities. We demonstrate observations for P- and S-waves incident parallel and perpendicular to the cable, and that cable coupling to the surrounding medium exerts far more control on signal quality than incidence azimuth. Compared to the nearest land station, an EW signal for a Mw5.8 earthquake was triggered 1.59 seconds earlier using P-wave arrivals on the fibre, and 3.89 seconds earlier using S-waves. We show that effective warnings can be made despite spatially decimating data by factors of 10 and 100 for P-waves and S-waves, respectively. This data volume reduction allows DAS on legacy seafloor cables to be integrated with EW systems for offshore earthquakes and other geohazards which threaten coastal populations.

## Introduction

The rapid increase in the use of fibre-optic sensing methods has had broad implications for seismological and earthquake monitoring. Distributed acoustic sensing (DAS) on optical fibres is an increasingly popular technology which enables dynamic measurements of differential strain along a cable^1^, offering spatial resolutions on the order of metres^2^. Sensing ranges can extend to over 100 km from a laser interrogator which detects the dynamic differential strain along the fibre^3,4^, allowing DAS to be applied for a range of applications including: monitoring ocean dynamics^5,6^, seismic imaging^7–10^, tracking marine activity^11–13^, and earthquake detection^14–16^. Critically, DAS can leverage pre-existing fibre-optic infrastructure such as telecommunications and some hybrid power cables (which integrate electrical conductors and optical fibres), reducing deployment costs and expanding monitoring capabilities offshore.

Despite these promising characteristics, the full integration of DAS into reliable real-time earthquake monitoring and early warning (EW) systems is an outstanding challenge, primarily for two reasons. First, the processing and storage of the immense volumes of data produced by DAS systems - typically multiple terabytes per day when recording continuously - presented a computational and logistical obstacle for previous works which analysed full DAS datasets^17,18^. The expanding network of seafloor cables presents substantial opportunities for integration into EW systems, but the data challenges of instrumenting them with DAS currently limits widespread implementation. Although traditional seismic^19–21^ and/or empirical^22–26^ denoising methods improve the signal quality, this comes at the expense of increasing the computational complexity of real-time monitoring. Second, the pronounced variability in DAS amplitudes for environmental signals - arising from spatial differences in the fibre–medium coupling and wave incidence directions - poses significant challenges for amplitude-dependent analyses, including moment tensor inversion and attenuation modelling^27,28^. This imposes significant challenges on the use of compressed-domain, feature-extraction based approaches^29^, especially over longer offshore distances where cable orientation relative to the ambient environment changes in both space (due to changes in bathymetry and horizontal azimuth) and time (with environmental dynamics), which in turn exacerbates the variability in DAS signal amplitudes.

**Fig. 1:**
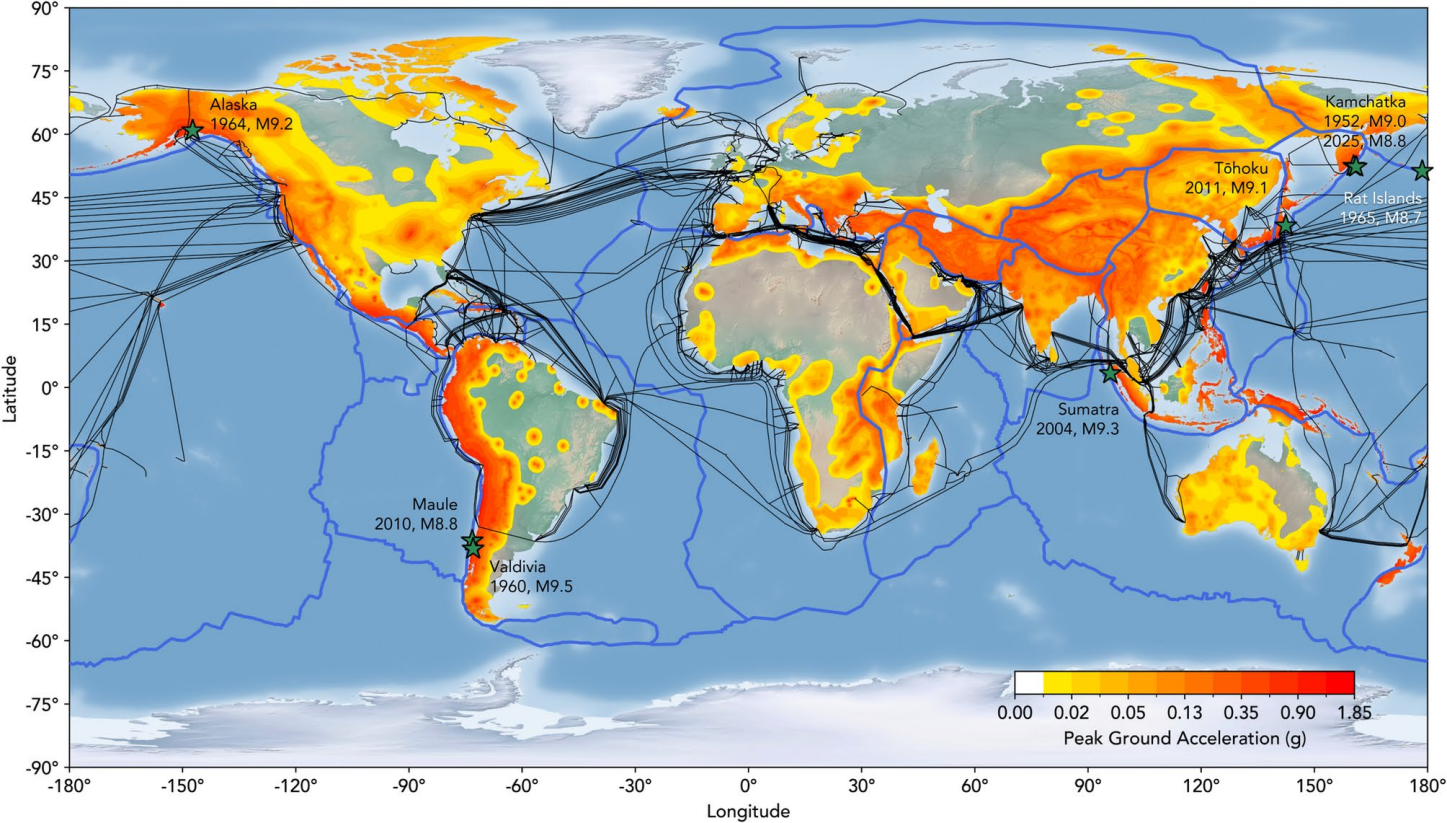
World relief map overlain with tectonic plate boundaries (blue), submarine telecommunication cables^51^ (black), and a geographical distribution of the peak ground acceleration with a 10% probability of being exceeded in 50 years, computed for reference rock conditions (the average shear wave velocity, Vs30, for the upper 30 m depth of 760–800 m/s)^52^. The largest offshore earthquakes in the last century are illustrated by green stars and labelled. Figure created using matplotlib in Python^53^.

The urgency of finding solutions to these challenges is emphasised by the global distribution of seismic hazards. As illustrated in Fig. 1, many of the world’s zones of highest hazard are populated coastal regions, where the nearest tectonic plate boundaries are typically located offshore. Earthquakes that occur near these densely populated coastal areas, as well as secondary geohazards including tsunamis, seafloor landslides, and coastal liquefaction, can have catastrophic human impacts, often leading to loss of life, widespread destruction of infrastructure, and the loss or disruption of essential services. Consequently, the implementation of effective and reliable early warning systems is crucial to provide seconds or minutes of notice, enabling people and systems to take mitigating actions prior to the arrival of seismic waves, for example, exiting buildings, taking cover, or automatically shutting down or protecting transport and power networks, and industrial sites.

The reduced coverage of offshore seismometers that contribute to EW systems means that warnings are only triggered once the nearest ground-based system detects an earthquake, and once a warning is verified for distribution. This issue is particularly acute in locations where large coastal population centres are situated between highly seismically active regions and the nearest seismic stations, severely limiting the availability of early warnings for offshore events. It is in these localities, however, that distributed acoustic sensing presents a transformative solution. Many of these high-risk areas are near a dense network of submarine cables which have the capability to be interrogated from onshore using DAS, and therefore leveraged for early warning systems. The potential of this existing infrastructure is particularly evident in regions like the Philippine and East China Seas, which have dense cable coverage connecting Asian hubs and Pacific Island groups, coinciding with the highly active Pacific, Philippine, and Australian plate boundaries. Similarly, cables off the coasts of North and South America propagate out from key locations along the coastline, providing dense linear arrays of sensors optimally positioned to detect offshore seismic events (Fig. 1).

To capitalise on this potential, it is critical to understand the factors which affect a DAS system’s response to incident seismic waves. Furthermore, an efficient method to assess such properties of a cable will determine those which provide the best signal quality, reducing the number of cables which need to be interrogated, further reducing the logistical challenges of working with DAS. In addition to the cable’s coupling to the seafloor, the arrangement of fibres within a cable, the presence of exterior armouring (common on submarine cables), the geometry of the cable itself, and its orientation relative to the seafloor can all impact the measured strain^30–34^. The interrogation method of the optical fibres dictates that the first-order response from environmental effects on the fibre are generated from deformation parallel to the cable, while perpendicular deformation creates only smaller amplitude second-order effects^35^. This theoretical understanding suggests that the orientation of a submarine cable relative to a seismic source is vital, but a key finding of this study contradicts that expectation. Complementary to reference 30, which showed that DAS fibres exhibit sensitivity to perpendicularly arriving waves from artificial sources on land^30^, this work demonstrates the same for perpendicularly arriving P- and S-waves from earthquakes on submarine cables. We find that local coupling plays a much stronger role in controlling DAS signal quality for earthquake monitoring than fibre geometry alone.

**Fig. 2:**
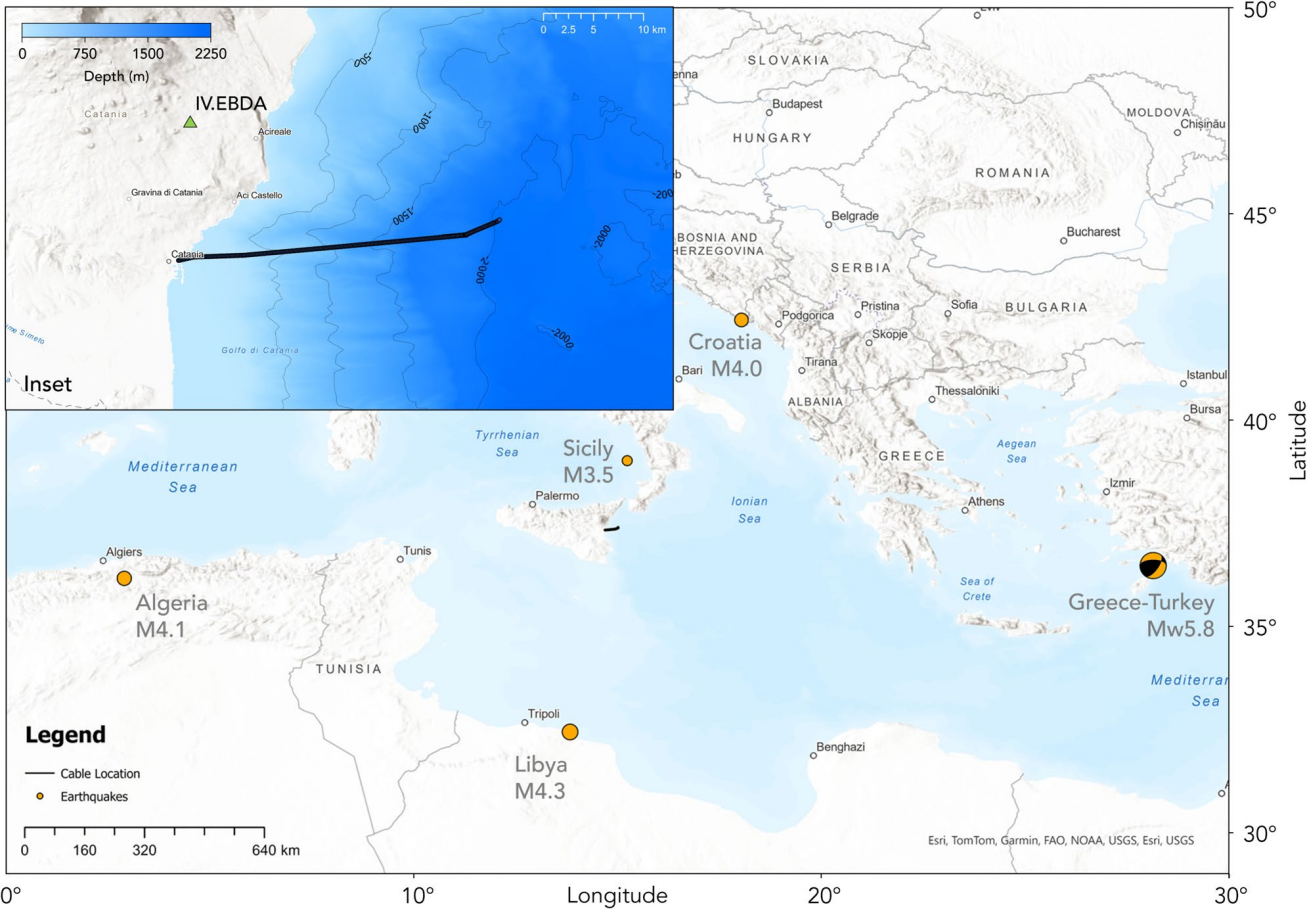
Map showing the approximate location of the fibre, and earthquakes used in this work. Background: terrain map with cable location (black) and earthquakes (orange) sized and labelled by magnitude and location as referred to in this work. Inset: position of the fibre from the landing station in Catania, Sicily, and location of the nearest land station (IV.EBDA; green triangle) overlain on contoured bathymetry^54^. Figure created using ArcGIS, see lower-right of figure for attribution.

Furthermore, typical requirements for early warning algorithms use P-wave or peak ground-motion observations to determine alerts^36^. Given the strain sensing approach of DAS, a robust body wave picking algorithm is needed which can operate on DAS data in real time, despite the aforementioned data volume challenges. This study investigates the viability of an ocean-bottom DAS fibre to act as an earthquake early warning system through the automated detection of P- and S-waves, contributing to a proof-of-concept. In particular, the impact of cable coupling, fibre geometry, and orientation are assessed through the detection of regional earthquakes, and their body wave arrival times are used to quantify early warning performance. Source mechanism estimation is also an important part of early warning systems to quantify the risks posed to people and infrastructure, and thus contributes to whether a warning is sent, though this work focuses specifically on computing early warning times and understanding factors which impact this quantity. Additionally, to address challenges of data volumes associated with DAS, these analyses are repeated after the data have been decimated spatially, to examine how the performance of DAS as an early warning system is affected by a reduction in the data flux. Analyses are performed on a dataset acquired using a bespoke-engineered optoelectronic interrogation system^37,38^ which was operated in a single ended optical interrogation of the fibre in a pre-existing seafloor cable at the Western Ionian Sea (WIS) EMSO Regional Facility in Catania, on the eastern coast of Sicily, Italy between May and June 2025. The 30 km fibre traverses 21 km offshore to depths of 2000 metres, with its approximate route to the ocean bottom observatory shown in Fig. 2. Data were collected at 1 kHz across 12,100 channels, each separated by 2.45 m with a gauge length of 4.90 m, before a temporal decimation to 10 Hz for data analysis. A bathymetric profile along the cable route is provided as Supplementary Figure S1 online. We investigate the azimuthal sensitivity of this predominantly East-West oriented fibre and determine the system’s capabilities for early body wave detection using five regional earthquakes which occurred during the deployment. These earthquakes were chosen with varying magnitude and bearing relative to the cable. We show that analyses of such short-term deployments with a handful of large events are useful to inform decision-making about the choice of cables which can be used for long-term tests and eventual integration into early warning networks.

## Results

### Earthquake observations

**Fig. 3:**
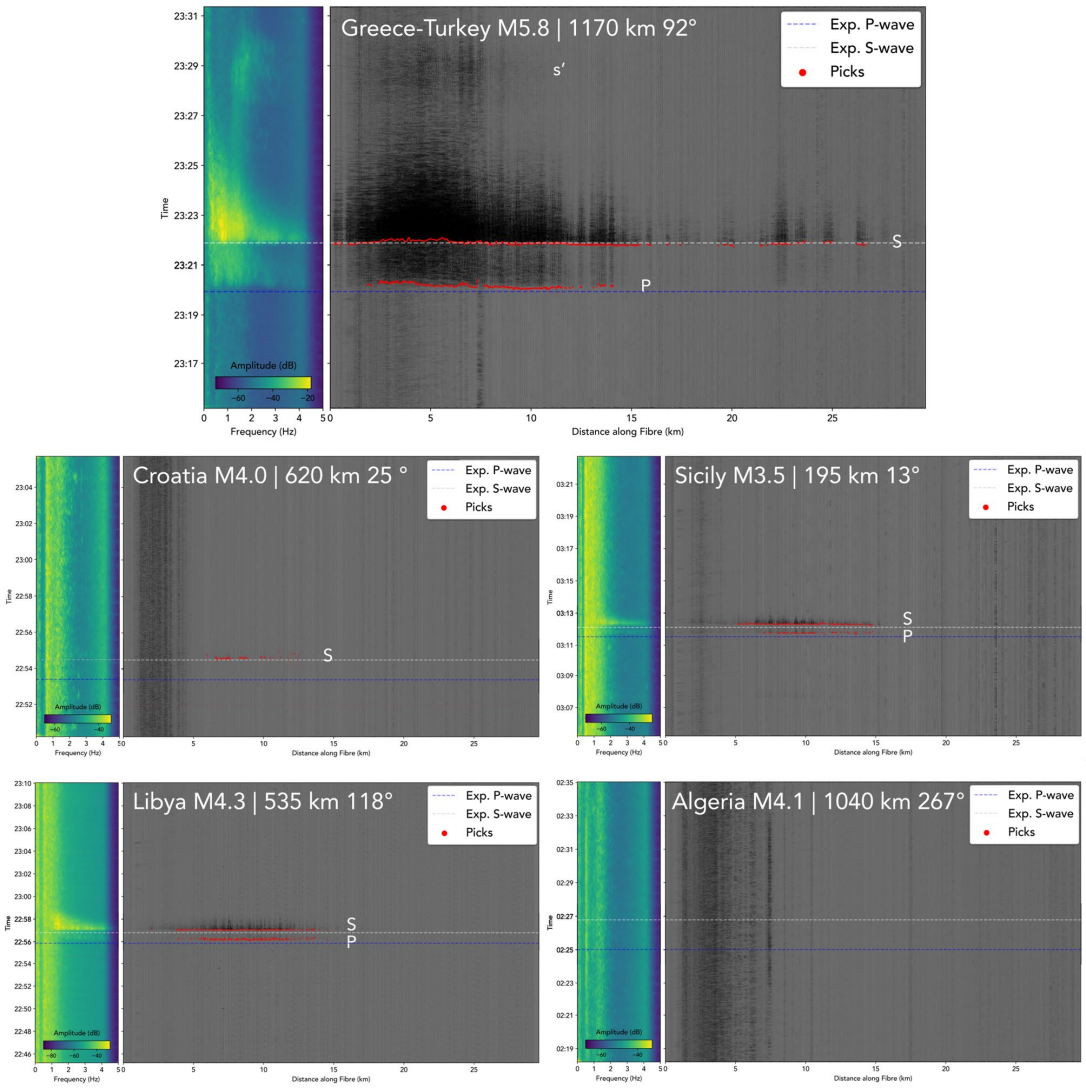
Illustration of P-, S-, and surface wave arrivals (labelled P, S and s’, where observed) along the full length of the fibre-optic cable for each earthquake analysed. From top to bottom, left to right: Mw5.8 Greece-Turkey, M4 Croatia, M3.5 Sicily, M4.3 Libya, M4.1 Algeria; each is labelled with the distance and bearing of the epicentre to the fibre. Left panels: average of the set of spectrograms for all channels. Right panels: recorded seismic traces at channels along the fibre measured from the interrogator and starting prior to the earthquake origin times. Red dots show the picked arrival times of P- and S-waves and blue and white dashed lines represent the expected arrival times for P- and S-waves, respectively, from a 1D spherically symmetric background model^55^.

The signals recorded at each channel of the WIS fibre for each of the five regional earthquakes (labelled with location and magnitude) are shown in Fig. 3. For the largest earthquake, a magnitude 5.8 event near the Greece-Turkey border, P- and S-waves are clearly observed in both the spectrogram and processed waveforms, with propagation velocities of $7.42\pm 0.44$ and $4.44\pm 0.32$ km/s, respectively. These observations are particularly prominent between 2 and 15 km, with surface waves also detected approximately eleven minutes after the earthquake between 3 and 7 km along the cable. As illustrated in the figure, the P- and S-wave arrivals are consistent with those expected from a 1D spherically symmetric background model^55^, and the average S-wave arrival is 1.49 seconds ahead of those predicted. Measured P-wave arrivals in the first half of the fibre trail those predicted by approximately 5 seconds, and sparse, less confident picks in the second half of the fibre differ by less than one second to those predicted.

The observed variations in arrival amplitudes along the fibre are attributed to a combination of factors. First, arrivals from this event propagate axially along the fibre’s East-West ($85^\circ$ bearing) orientation, providing a near-optimal $9^\circ$ azimuth of incidence for body wave strain sensing. As the fibre curves northward towards the end of the fibre, incident wavefronts arrive more perpendicularly to the fibre $50^\circ$which is expected to reduce P and SH wave recorded amplitudes. Second, the far end of the fibre is likely affected by the complex bathymetric profile of the region, with a narrow continental shelf dropping steeply into the Ionian Sea as part of the Malta Escarpment. Up to 6 km along the fibre, the gradient of the seafloor is no more than 3 degrees, allowing an accumulation of sediment which provides strong coupling between the fibre and seabed. Beyond this point, the seafloor gradient increases and varies dramatically, which is expected to lead to a rockier substrate and much weaker coupling, while also increasing the likelihood of suspended sections of fibre coupled only to the surrounding water.

**Fig. 4:**
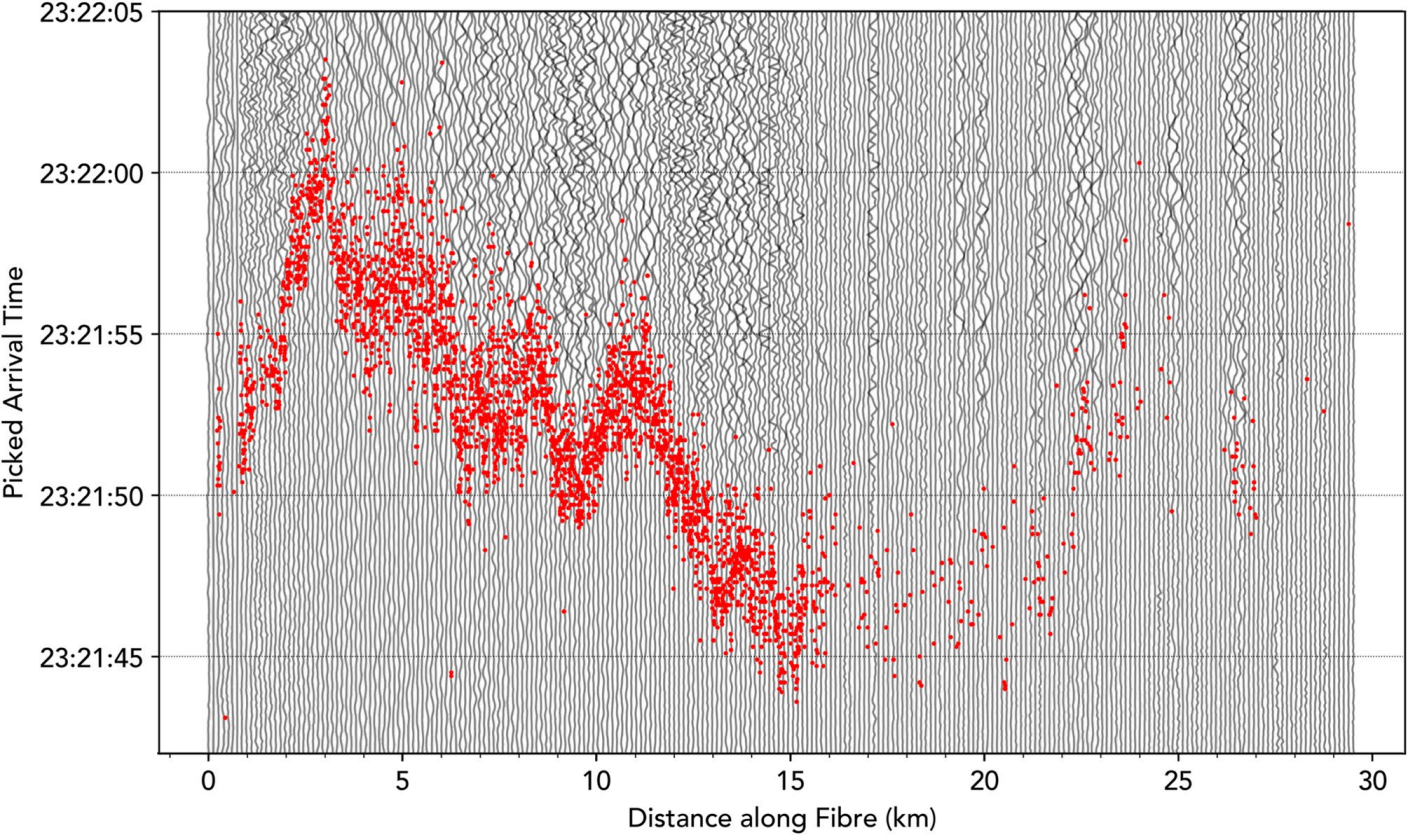
Picked S-wave arrival times with signal-to-noise ratios above 3, for the Mw5.8 Greece-Turkey earthquake overlain on traces from every 50th DAS channel for reference. Most arrivals are detected in the first 15 km of the fibre, but the variation in the arrival times due to velocity changes beneath this section is up to half as large as any approximately linear increase due to increased travel distance of the incident waves.

Fig. 4 highlights the S-wave arrival times for this Mw5.8 event and illustrates the variation in arrival times leading up to the cable landing station. In theory, waves travelling at a constant velocity along a straight fibre would display a linear moveout relative to their propagation speed, which might be expected to be observed at least approximately for surface waves travelling along the azimuth of the cable. However, in practice this is complicated by the non-linear cable geometry and elevation changes, as well as external factors such as inhomogenous media, variable coupling, and significant changes in the propagation paths of refracted body waves observed in different parts of the cable. The arrivals in the first 15 kilometres of the fibre, where the cable is laid in a straight line and body waves from this event are incident on the fibre at an azimuth angle of approximately $9^\circ$, vary with a complex, non-linear moveout. This is attributed to the local velocity structure beneath the cable, with variable low-velocity offshore sediment controlling the arrival time delay. The 10-15 second variation in arrival times between 15 and 3 km along the fibre corresponds to an S-wave propagation velocity of between 1.2 to 1.5 km/s, which is consistent with sedimentary rock^39^. Such delays caused by local velocity structures need to be known and accounted for in EW networks, perhaps by only using picks from lengths of the fibre overlying thinner or faster sediments, which cause reduced delay times.

In addition to bathymetry, the near-shore section of fibre exhibits a reduced sensitivity to seismic energy which is attributed to the construction and deployment of the fibre. The first 1.6 kilometres of fibre is doubly armoured, while the following span to 13 km is single-armoured, and the remainder protected only with lightweight armour. Lightweight armour typically consists of optical fibres surrounded by copper tubing, plastic (e.g., polypropylene), and layers of armoured wires (often galvanised steel) wrapped in nylon ropes^30^. Singularly and doubly armoured cables then have additional layers of larger diameter, armour wires and surrounding plastic. Given the increased risk of cable breaks on near shore fibres^40^, the cable is also likely buried into sediment in the shallow waters near the landing station for better protection against anchors and the activities of shipping and fishing vessels, such as trawling^41^. This will further impact the amplitude of signals observed in this portion of the fibre, compared to more weakly coupled channels further eastward.

The travel-time picks shown in Fig. 3 are determined following the implementation of an automated body wave picking algorithm (see ‘Methods, Arrival and SNR Picking’). Some S-wave picks are made towards the offshore end of the cable which may coincide with small plateaus in the bathymetry where the cable may have spatial intervals over which it is more strongly coupled. The second event whose body waves travelled parallel to the fibre was a magnitude 4.1 earthquake in Algeria on the 5th of June, a similar distance (1,040 km) from the fibre as the Greece-Turkey 5.8 event (1,170 km). No observation of this event could be made on the cable, as was the case for nearby land stations. This is attributed to the significantly lower magnitude of the event, but could also be affected by the source mechanism and depth of the earthquake. It does, however, provide a lower bound of detectability for the system.

Contrary to the expectation that perpendicular arrivals would be poorly resolved, particularly for compressional P-waves, all three remaining events were observed on the fibre despite northerly or southerly bearings. Both P- and S-waves were detected for both a 292 km deep M3.5 event 195 km North of the fibre ($13.1^\circ$ bearing from the fibre), and a M4.3 event at 25 km depth, 535 km South of the fibre ($188.7^\circ$ bearing). Only weaker S-waves were detected for a magnitude 4 event 620 km Northeast ($25.12^\circ$ bearing) in Croatia. All automated picks of these arrivals are within the first 15 km of the cable, which is orientated almost perpendicularly to the incident waves, with body wave incidence azimuths ranging from 59 to $76^\circ$ in this part of the fibre. In line with the theoretical $\cos ^2(\theta )$ directional sensitivity of DAS to P-waves, arrivals incident at these angles should have up to a 17 times weaker response than those axially incident. S-wave sensitivity is higher than P-waves, with a contributions of $\sin (2\theta )$ and of $\cos ^2(\theta )$ for larger incidence azimuths such as those observed here, as opposed to the $\sin (2\theta )$ term which dominates for smaller angles. This suggests that the stronger coupling in this section of the fibre is a far more critical factor for earthquake detection than the geometrical orientation of the fibre itself, with the terminal portion of the fibre $41^\circ$providing no observations of any of these three events, despite having the most favourable incidence azimuths *a priori*.

Applying the same picking algorithm to the data from the nearest land station for all five events, only the two largest magnitude earthquakes had sufficient signal-to-noise (SNR) ratios to be detected (see ‘Methods, Arrival and SNR Picking’ for an explanation of SNR computation). For the Libya earthquake, the fibre and land station are at approximately the same latitude and so neither provides a significant early warning signal. P-waves from the Mw5.8 earthquake, however, were detected 1.59 seconds earlier on the fibre than on land, and S-waves 3.89 seconds earlier, due to the fibre’s location between the epicentre and land station. A longer fibre would be expected to increase these time advantages linearly, given favourable coupling.

### Spatial decimation

**Fig. 5:**
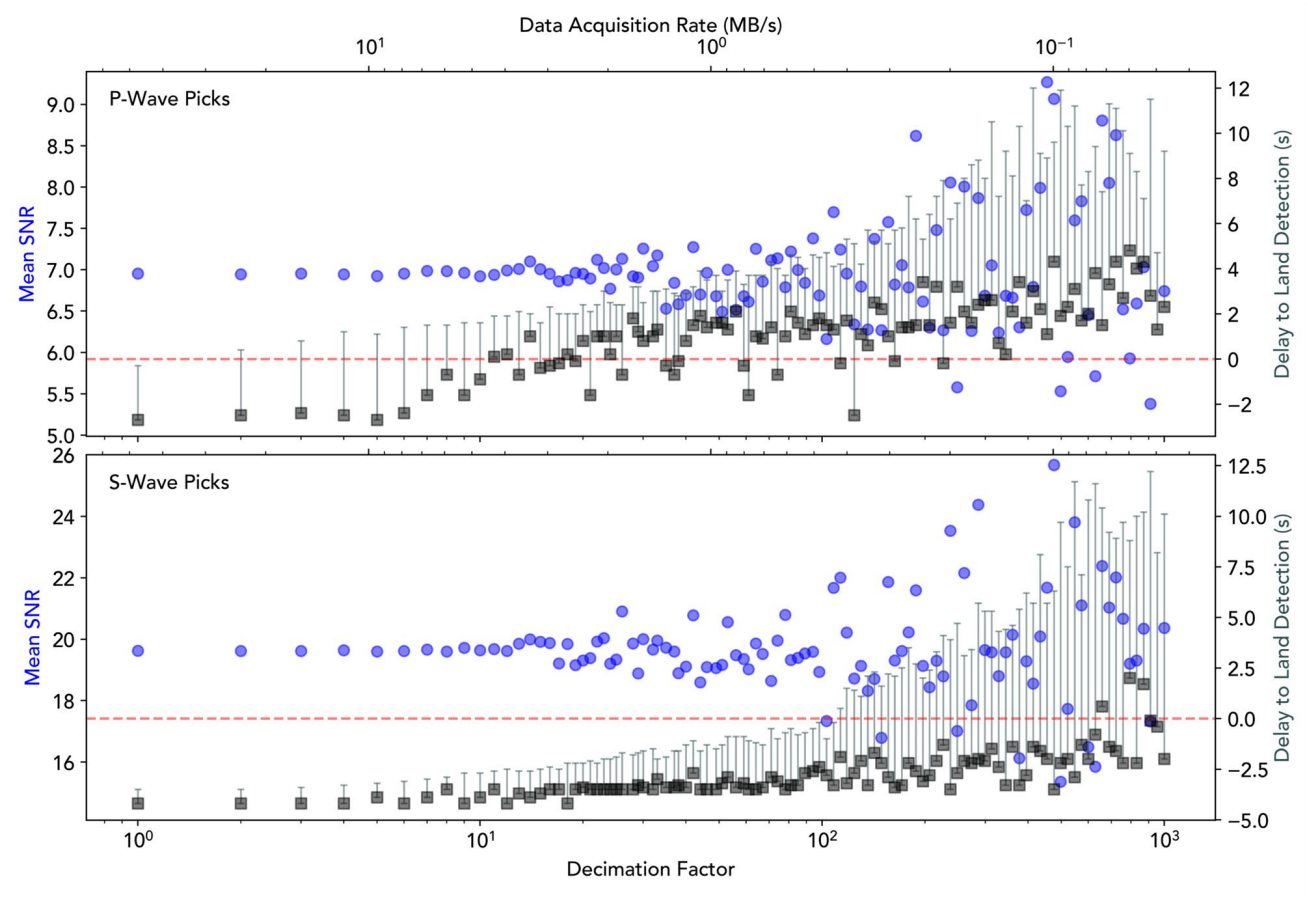
Impact of spatial decimation on the signal-to-noise ratio of P-wave (upper panel) and S-wave picks (lower panel) and the amount of early warning compared to the nearest land station (IV.EBDA) for the Mw5.8 earthquake. Abscissae: the factor of spatial decimation (bottom axis) and the corresponding effective data acquisition rate in MB/s, assuming 1 kHz acquisition (top axis). Left ordinates: mean signal-to-noise ratio of the picked arrivals relative to the background noise of each channel (blue circles). Right ordinates: the time between the land station pick (red dashed line) and DAS picks; black squares represent the earliest arrival time and error bars show the range within which the earliest 10 arrivals were detected.

The analysis above was repeated while the recordings are increasingly decimated spatially to reduce data size. We define a decimation factor which selects every $\text {N}^\text {th}$ channel from the DAS records; a decimation factor of 1 corresponds to the original data, comprising one channel every 2.45 metres - a total of approximately 27 GB of data every 10 minutes. Fig. 5 shows the impact of spatial decimation on the mean SNR of all picks, and the amount of early warning provided as compared to the nearest land station for the 5.8 Greece-Turkey magnitude earthquake. The mean SNR ratio of both pick types remains steady for decimation factors approaching 50 to 100 (which would reduce the 27 GB above to 0.27-0.54 GB) since even large decimation factors result in many picks due to the high spatial resolution of DAS. Due to the higher amplitude of incident S-waves, an early warning signal could be produced earlier than on the nearest land station even with a decimation factor of over 100. For lower amplitude P-waves, the maximum decimation factor which still provides an early warning is reduced significantly to a factor of approximately 10 (90% reduction in data volume), seen where the grey arrival points intersect the $t=0$ red dashed line. This is because the P picks increasingly occur at the high SNR onshore end of the fibre. The sensitivity of P-wave detections to decimation could be improved by a more robust picking algorithm and data pre-processing to increase the signal-to-noise ratio of incident arrivals.

At very high decimation rates, the variance of both quantities is seen to increase. This is a result of the sample size being reduced to only a few tens of channels, of which only a handful might contain identifiable arrivals. Consequently, increasing decimation acts as a form of sub-sampling on a very limited number of channels, which will not provide reliable results due to variation in the coupling, orientation, and other physical factors affecting the fibre. Other data-informed volume reduction methods could be used, for example, selecting channels based on their signal-to-noise ratios however, this increases computational complexity as channels would not be immediately discarded, and this would also bias sections of the cable with favourable coupling, which may be closer to the shore and provide less early warning. Furthermore, the data acquisition rate reported in Fig. 5 is based on the original sampling frequency of 1 kHz, common for DAS deployments. However, if a fibre were interrogated solely as part of an early warning network, a sampling frequency of 100 Hz would be sufficient for detection of local, regional, and teleseismic events, further reducing the volume of data collected.

Our analysis emulates a case in which data would be spatio-temporally decimated as part of the acquisition process, or prior to storage, to reduce computational and storage requirements for real-time processing in EW systems. To test this in the real world, the DAS system was reconfigured during the deployment to record with a channel spacing of 10 m instead of 2.5 m, acting as a decimation factor of 4. This configuration was in effect for the Sicily and Libya earthquakes, which were both well detected, despite the reduction in data flux. In particular, P-waves from the Libyan event were detected with this decimation factor, while they were not seen following the Croatian earthquake which was similarly off-axis from the fibre’s orientation, and only 85 km farther away, and 0.3 magnitude units lower. This suggests that the attenuation of seismic energy as it propagates (and the intensity of the earthquake itself) are more detrimental than the decimation factor for detecting P-wave arrivals.

## Discussion

This study demonstrates that a typical seafloor fibre-optic cable interrogated with distributed acoustic sensing can provide robust observations of P- and S-waves from regional earthquakes, as well as surface waves from those of large magnitude. Despite using a regional earthquake in the Mediterranean, for which an early warning for the regions with the most intense shaking would not be provided by a cable in this location, this work demonstrates a proof-of-concept for using DAS arrays to provide early detection of events in remote and coastal regions where seafloor fibres would naturally be positioned so as to provide early warning insights. A key finding is that the coupling of the fibre to its surroundings is a more critical factor for body wave detection than the incidence azimuth at which these waves arrive on the fibre. This suggests that pre-existing submarine cables, regardless of their orientation provide a good opportunity to be repurposed for extensive earthquake early warning networks.

The compelling observations of the Mw5.8 Greece-Turkey earthquake underscore the significant potential of DAS in EW systems. The geometry of the interrogated fibre, which propagates perpendicularly outward from a coastal city, enabled the detection of incoming body waves for a regional earthquake, which could have triggered an early warning alert. Although this particular event was detected by a wealth of land stations across the Aegean before reaching Sicily, this configuration mirrors that of many other submarine cables (Fig. 1), which are often laid perpendicular to the shore to connect countries and cities to international cable networks. Particular sites where existing fibres are well suited for potential DAS EW systems in high seismic hazard regions include cities along the western coasts of North and South America, as well as Japan, Taiwan, the Philippines and other regions of Southeast Asia. By leveraging this existing infrastructure, DAS offers a transformative and cost-effective solution to extend seismic monitoring coverage and improve the accuracy and timeliness of alerts, particularly in seismically active regions with sparse permanent seismic station coverage. This solution is easily scalable to a large number of submarine cables, and having a global network of fibres would enable additional characteristics of earthquakes to be determined such as their epicentres^42^ and ground shaking intensities^43,44^, further informing early warning signals.

While the results also reveal limitations in detection range, as shown by the lack of observations for the Algerian earthquake, and minimal S-wave detections for the Croatian event, this does not impact the performance of DAS as an early warning system. The primary goal of such a system is to issue warnings for large earthquakes with high destructive potential, rather than cataloguing all events (though DAS cables can contribute to this goal^45^). These results show that events M>5.8 can be detected and warnings produced at epicentral distances exceeding 1000 km, and for those M>4.3 at least 500 km away, even when spatio-temporal decimation is actively applied. On a regional scale, these capabilities are more than sufficient to act as an early warning system for earthquakes which pose immediate dangers to population hubs which might otherwise receive no, or a much-reduced warning, with current systems.

By providing rapid strain measurements across large areas using existing cable networks, DAS can enhance real-time data acquisition and facilitate quicker decision-making processes, even when data are decimated to reduce the operational constraints of working with large DAS data volumes. Repurposing existing submarine cables using a DAS interrogator can provide an alternative and more cost-effective solution to the deployment of multiple land or ocean bottom seismometers (e.g., Japan’s S-net^46^) which incur a very high cost to implement and maintain. Short-term deployments such as this one can provide a method to quickly assess the signal qualities produced by the fibre using just a few seismic events, allowing more informed decision-making for future deployments and about which cables to integrate into EW networks.

Building on the results of this study, future work should focus on several key areas to facilitate the integration of DAS into operational EW systems. There is a need for the development of more robust and computationally inexpensive pre-processing and picking algorithms optimised for real-time body wave detection for DAS data. Other physical characteristics of the fibre, such as using helically wound fibre, varying armouring and trenching methods, and cable types (telecommunications, power) - all of which may vary on pre-existing cables - may impact detection capabilities. In addition to timely and accurate detection capabilities, early warning networks should include the ability to characterise earthquake source parameters; a single fibre can confine a tight arc segment for an epicentre location^42^, an L-shaped fibre or multiple orthogonal cables can be used to better constrain earthquake locations, particularly where the coupling of such orthogonal sections is of good quality.

It is also necessary to quantify the limitations of the acquisition system more precisely. This would involve collecting data from cables in other high-risk regions - such as the west coast of the Americas (one such example has already shown promising results^18^) or Southeast Asia - to better constrain the minimum (and maximum, due to sensor saturation or phase-unwrapping limits) detectable earthquake magnitude, maximum epicentral distance, and the corresponding early warning time provided. The active integration of these methods into an existing early warning network could provide a compelling argument for a more widespread deployment. Such efforts would provide the necessary framework for turning the potential of DAS into a practical, reliable, and globally deployable seismic early warning solution.

## Methods

### Data acquisition and processing

Originally sampled at 1 kHz, the data were first decimated to 10 Hz for 15–20-minute spans of the time periods of interest using overlapping windows. There is no defined instrumental response for DAS interrogators, but it has been shown to be approximately flat over a broad frequency range^47^. Previous studies have used nearby seismometers to calibrate DAS measurements^48^, however the nearest seismic stations to this fibre are on land in Sicily, and therefore do not provide a representative comparison. This work highlights that early warnings could be obtained with far less calibration than is achieved in more conveniently designed previous studies. The interrogator used in this experiment measures the differential change in the phase, $\Delta \phi$, of the backscattered Rayleigh signal, normalised by the gauge length, $G_\text {L}$. Such raw amplitudes are often converted to strain, $\varepsilon$, however we argue that the normalised differential phase change is a more appropriate and representative metric. Conversion to strain is in principle achievable through the equation

1 $$\begin{aligned} \frac{\Delta \phi }{G_\text {L}}\frac{\lambda }{4\pi n_\text {eff}z}=\varepsilon \end{aligned}$$

where $\lambda$ is the wavelength of laser light used, $n_\text {eff}$, the effective refractive index in the fibre, and *z* is the photoelastic correction factor which reduces the measured phase to account for the refractive-index change partially opposing the geometric elongation. The refractive index of the cable varies as a function of temperature, which in this study site can vary by up to about four degrees Celsius between sea surface and sea bed, in addition to daily, seasonally, and as a result of other temporal trends and environmental conditions, thus affecting the strain conversion. Furthermore, the Rayleigh backscattering measurements will capture such temperature fluctuations as a function of frequency. In the 1-5 Hz range of interest in this study, the temperature variations measured will be orders of magnitude lower than the bulk $4^\circ$C surface-seabed temperature difference and their contribution to the refractive index can be considered static. However, for much lower frequencies, which may be used for interferometric or inversion applications, thermal factors will become important and the strain conversion from differential phase change will not be valid. This study maintains differential phase change measurements throughout in light of this discussion, but strain values can be obtained with the linear conversion factor of $\varepsilon =1.0637\times 10^{-7}\Delta \phi /G_\text {L}$ ($z=0.79$, $\lambda =1550.12$ nm, $n_\text {eff}=1.4679$). The self-noise of the system as reported by the manufacturer is approximately 0.8 to 0.9 pico-strain per root Hz at 100 Hz and tested with a 10 m gauge length.

Furthermore, the analyses presented in this work are mostly kinematic, picking arrival times without estimating physical properties from amplitudes, and so removal of an instrument response is not necessary. Instead, linear and mean trends were subtracted from each channel to correct for any long-period instrumental or environmental drift, before all channels were normalised. Extracting seismic energy from the data was done by filtering in frequency-wavenumber space with a velocity interval of 200 m/s to 10 km/s with a taper of 50 and 500 m/s at the lower and upper bounds, respectively. In practice, real-time f-k filtering may affect the data quality, particularly since a small sliding window will likely be required for the desired processing latency which would reduce spectral resolution and may cause spectral leakage. Alternative approaches (such as machine learning) to body wave detection may obviate the use of f-k filtering. Similarly, different cable deployments will also impact the amount of data processing required, depending on the coupling and overall sensitivity of each unique DAS system to body waves.

### Arrival and SNR picking

To automatically pick body wave arrivals, a peak-finding algorithm is implemented using a short-term average/long-term average (STA/LTA) trigger. Increases in the STA/LTA occur when energy from a short-term event exceeds that of a baseline background noise power, providing the timing of changes in a time series. To further improve this method, the STA/LTA signal is smoothed using a Gaussian filter to reduce the algorithm’s sensitivity to noise, before its derivative is computed. Arrivals are picked as peaks in this derivative which exceed a predefined threshold. This technique identifies the moment of the maximum change in the energy between the short- and long-term averages, which is a more precise indicator of a wave’s arrival than the STA/LTA trigger alone. The same parameters used in this process were used throughout the picking process for all varying decimation degrees, which could affect picking performance. More complex picking algorithms are available, such as PhaseNet-DAS^49^ which may improve the extraction of wave arrivals, as would a more refined data processing approach, but our method has been created to be computationally rapid and inexpensive to allow it to run in real-time on a large DAS dataset. PhaseNet-DAS has been recently demonstrated in real-time operations on heavily spatially decimated DAS data^50^.

To remove spurious picks, the spatial density of DAS channels is exploited to find peaks in the distribution of the picked arrival times above a threshold. P- and S-wave arrivals are observed as two peaks in this distribution. Provided these peaks exceed a predefined threshold, arrivals within the peaks’ widths (width at 95% relative height) are accepted as arrivals. The signal-to-noise of each pick was computed as the ratio of the root-mean-square (rms) amplitude of the signal in a 60-second window following a pick (or the duration of the gap between P and S arrivals, if smaller than 60 seconds) to the rms amplitude of an 80-second section from the start of each trace prior to any arrivals. The time series used for these analyses were chosen to ensure sufficient background noise prior to any body wave arrivals. Peaks were then rejected if their signal-to-noise ratio was below 3.

## Supplementary Information

Supplementary Information.

## Data Availability

The datasets generated and analysed during the current study are available in a Zenodo repository: https://doi.org/10.5281/zenodo.17571118. Seismic data from station IV.EBDA is also available from the same repository.
