# Supplementary material for: Azimuthal sensitivity and spatio-temporal decimation of data from distributed acoustic sensing on submarine cables for offshore earthquake early warning systems
Source: Sci Rep. 2025 Nov 25;16:7. doi: 10.1038/s41598-025-29234-5 (PMC12765012; doi:10.1038/s41598-025-29234-5)
Supplement: Supplementary file 1 — Supplementary Information. [file 41598_2025_29234_MOESM1_ESM.docx]

**Supplementary Material for ‘Azimuthal Sensitivity and Spatio-Temporal Decimation of Data from Distributed Acoustic Sensing on Submarine Cables for Offshore Earthquake Early Warning Systems’ by Smith *et al.***


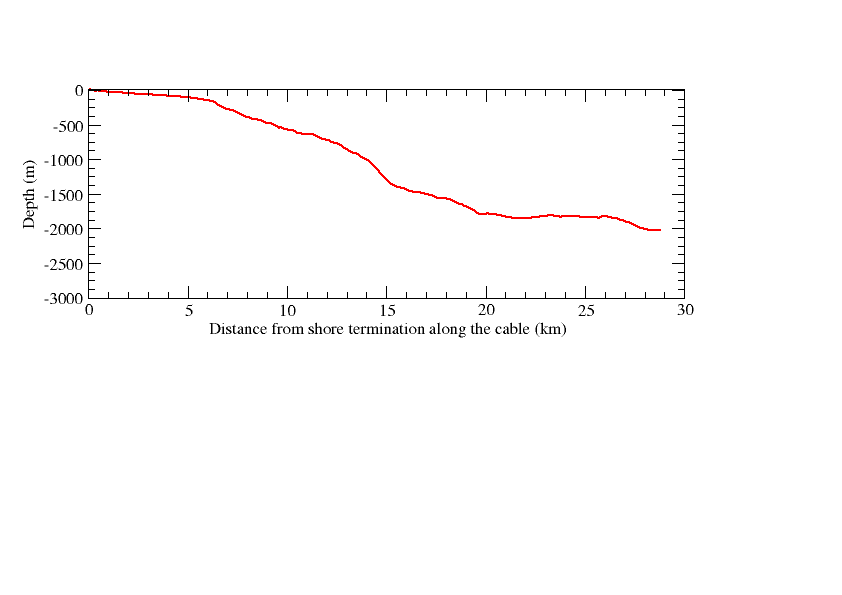
**Supplementary Figure S1**

Supplementary Figure S1: Plot of cable profile with bathymetry, produced using EMODnet^1^.

**References**

[1] EMODnet Bathymetry Consortium. EMODnet Digital Bathymetry (DTM 2024). EMODnet <https://doi.org/10.12770/cf51df64-56f9-4a99-b1aa-36b8d7b743a1> (2024)
